# Microsurgical treatment of middle cerebral artery stenosis or occlusion: a single center experience and literature review

**DOI:** 10.1186/s12893-022-01539-6

**Published:** 2022-03-07

**Authors:** Li Zhang, Congyan Wu, Handong Wang, Lei Mao

**Affiliations:** Department of Neurosurgery, Jinling Hospital, School of Medicine, Nanjing University, 305 East Zhongshan Road, Nanjing, 210002 Jiangsu People’s Republic of China

**Keywords:** Middle cerebral artery stenosis or occlusion, Clinical characteristics, Microsurgery, Vascular bypass

## Abstract

**Background:**

To investigate the effect of superficial temporal artery-middle cerebral artery (STA-MCA) bypass in the treatment of MCA stenosis or occlusion.

**Methods:**

The clinical and imaging data of 31 MCA stenosis or occlusion patients with STA-MCA bypass were analyzed retrospectively. The operation was performed by STA-MCA M4 segment bypass via the frontotemporal approach. Modified Rankin Scale (mRS) was used to evaluate the neurological function of patients.

**Results:**

After operation, head computed tomography (CT) showed that there was no new infarction or hemorrhage in the operation area. CTA and CTP showed that the bypass vessel was unobstructed in 29 cases and the cerebral perfusion was improved in 31 cases. Among the 31 patients, 7 patients had postoperative complications and 13 patients had improvement of clinical symptoms. The other patients had no complications and the clinical symptoms remained unchanged. The mRs score of 31 patients after operation indicated that the neurological function was significantly improved than pre-operation. Of the 31 patients, 23 cases were followed up. The mRs score showed that the neurological function of these 23 patients was further improved than that at discharge. In addition, DSA (or CTA) and CTP showed that the bypass vessel was unobstructed and the cerebral perfusion was further improved.

**Conclusion:**

STA-MCA bypass was an effective method for the treatment of MCA stenosis or occlusion. However, the results should be further verified by large sample, multi-center and long-term follow-up.

## Introduction

Middle cerebral artery (MCA) stenosis or occlusion is an important type of ischemic cerebrovascular disease, which is the late stage of intracranial atherosclerosis. Some patients may form collateral compensation in the progression of disease, however if the compensation is insufficient, patients may occur transient ischemic attack (TIA) or even stroke [1]. At present, the treatments of ischemic stroke include extracranial and extracranial vascular bypass, intravascular interventional therapy and drug therapy. For patients with MCA stenosis or occlusion, due to its hemodynamics disturbance, drug treatment can only achieve limited effects [2]. While intravascular interventional therapy is for specific patients, the segment and length of stenosis or occlusion are important for the choice of patients. In addition, the risk of early bleeding and thrombosis formation is high [3]. In comparison, the super temporal artery (STA)-MCA bypass is an end-to-side anastomosis of extracranial and intracranial vessels, which can rapidly increase cerebral blood flow, improve ischemic symptoms and nervous system function [4]. Currently, STA-MCA bypass has become the main surgical method for the treatment of MCA stenosis or occlusion. The purpose of this study is to explore the clinical characteristics and treatment strategies of patients with MCA stenosis or occlusion, so as to provide reference for clinical diagnosis and treatment.

## Material and methods

### Patient selection

All methods used in our study were performed in accordance with the Declaration of Helsinki and was approved by the Human Ethics Committee of institutional review board of Jinling Hospital. Written informed consent was obtained from individual or guardian participants. We identified 31 consecutive patients with MCA stenosis or occlusion in the Department of Neurosurgery, Jinling Hospital from August 2015 to August 2020. The clinical features of patients were shown in Table 1.

**Table 1:** Base-line characteristics of 31 MCA stenosis or occlusion patients

Clinical data	Value (31)
Sex	
Male	19 (61.29%)
Female	12 (38.71%)
Age (range)	(55 ± 6) years (43 ~ 65 years)
Courses (range)	30 (1, 90) days (1 h ~ 30 years)
Symptoms	
Dizziness	11 (35.48%)
Numbness and weakness	7 (22.58%)
Language dysfunction	7 (22.58%)
Dyskinesia induced by TIA	6 (19.35%)
History	
Hypertension	15 (48.39%)
Diabetes	8 (25.81%)
Diameter stenosis	
Severe stenosis (≥ 70%)	12 (38.71%)
Subtotal occlusion (≥ 90%)	6 (19.35%)
Total occlusion (100%)	13 (41.94%)
Anastomosis	
Obstructed	2 (6.45%)
Unobstructed	29 (93.55%)
Complications	
Poor healing of incision	1 (3.23%)
High perfusion	3 (9.68%)
Low perfusion infarction	3 (9.68%)
Improvement	
Limb muscle strength	3 (9.68%)
Language dysfunction	4 (12.90%)
Recurrent TIA	6 (19.35%)
Preoperative mRs score	
0	6 (19.35%)
1	12 (38.71%)
2	13 (41.94%)
Postoperative mRs score	
0	10 (32.26%)
1	15 (48.39%)
2	6 (19.35%)

MCA, middle cerebral artery; TIA, transient ischemic attack; mRs, modified Rankin scale

### Inclusion and exclusion criteria

Inclusion criteria: (1) imaging examination indicated the stenosis or occlusion of MCA in M1 segment; (2) the stenosis of MCA M1 segment > 70%, and the collateral circulation compensation was insufficient [5]; (3) patients still had recurrent TIA or stroke after maximal medical therapy; (4) mild to moderate neurological dysfunction, and the modified Rankin scale (mRs) score ≤ 2; (5) CTP showed that there were decreased cerebral blood flow and hemodynamic disorders in the blood supply area of stenotic or occluded artery; (6) patients were not suitable for intravascular interventional therapy because the blood vessel is excessively severe tortuosity or the plaque calcification is severe, and the stent cannot be placed; (7) informed consent of patients and their families.

Exclusion criteria: (1) age > 70 years old; (2) severe basic diseases, such as heart, lung and kidney dysfunction, diastolic blood pressure > 110 mmHg, fasting blood glucose (FBG) > 16.6 mmol/L; (3) patients had severe neurological dysfunction after medical therapy, such as large area cerebral infarction or internal capsule infarction, mRs score > 3; (4) STA was stenosis or thin, which was difficult to complete bypass; (5) although there was stenosis or occlusion of MCA, CTP showed that the cerebral blood flow of the stenotic artery was normal; (6) the patients or their families refused surgery.

### Surgical procedure

Before operation, all patients took single antiplatelet drug, aspirin, 0.1 g/day. The location of STA was marked by palpation. In the operation, the frontal and parietal branches of the STA (donor vessel) were dissociated and stained by methylene blue. After opening the dura, we chose the M4 segment of MCA (recipient vessel) with the same diameter as the distal part of STA. Then, we block the recipient vessel by two temporary aneurysm clips. Subsequently, the donor and recipient vessels were anastomosed end to side with 10–0 vascular suture under the microscope, each side was sutured with 3–4 needles. Finally, the aneurysm clips were released to confirm that the anastomotic site was unobstructed without bleeding. After operation, low molecular weight heparin calcium was injected subcutaneously temporarily to prevent anastomotic thrombosis, and then aspirin was taken orally, 0.1 g/day.

**Fig. 1:**
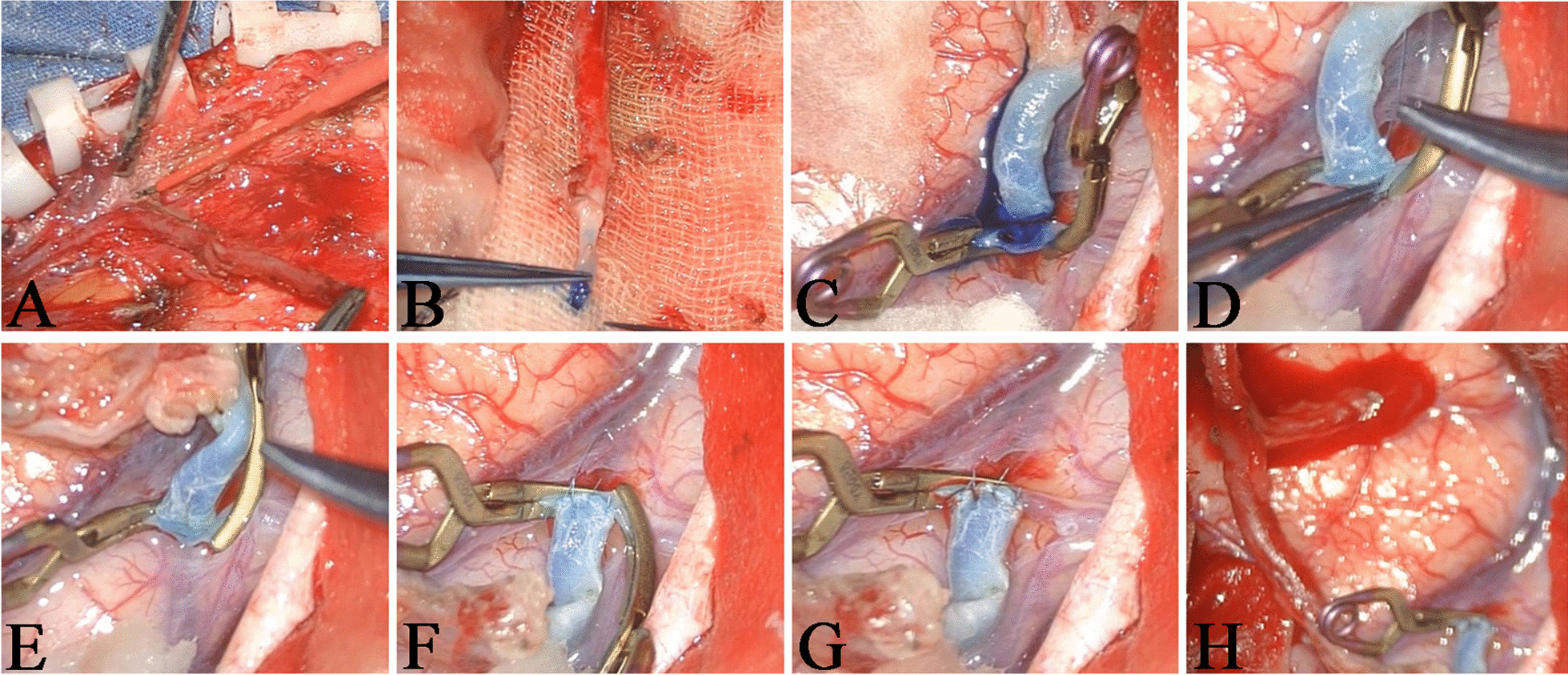
The surgical procedure of STA-MCA bypass. **A** The frontal and parietal branches of the STA were separated. **B** The parietal branch (donor vessel) was stained by methylene blue. **C** The M4 segment of the MCA (recipient vessel) was blocked by two temporary aneurysm clips and the vessel wall was cut. **D** The donor and recipient vessels were anastomosed end to side. **E** and **F** Each side of the vessels was sutured with 3–4 needles. **G** One aneurysm clip was released and there was no bleeding at the anastomotic site. **H** The blood reflux of frontal branch was well

Because the STA had frontal branch and parietal branch, we usually dissociated the two branches. If the diameter of the parietal branch ≥ 1 mm and the progress of bypass was well, we only performed the parietal branch bypass. If the diameter of the parietal branch < 1 mm or the progress of bypass was not well, both the frontal and parietal branches bypass were performed to increase the intracranial blood supply. The detailed surgical procedure for the STA-MCA bypass was shown in Fig. 1.

### Statistical analysis

Statistical analysis was performed using the SPSS 20.0 software. Continuous variables were presented as mean ± SD and the parameters were compared using the t test. Categorical variables were analyzed using the chi-square test. Skewness distribution variables were presented as medians and quartiles [M (P25, P75)] and the parameters were compared using the rank-sum test. A value of *P* < 0.05 was considered statistically significant.

## Results

### Preoperative imaging data

**Fig. 2:**
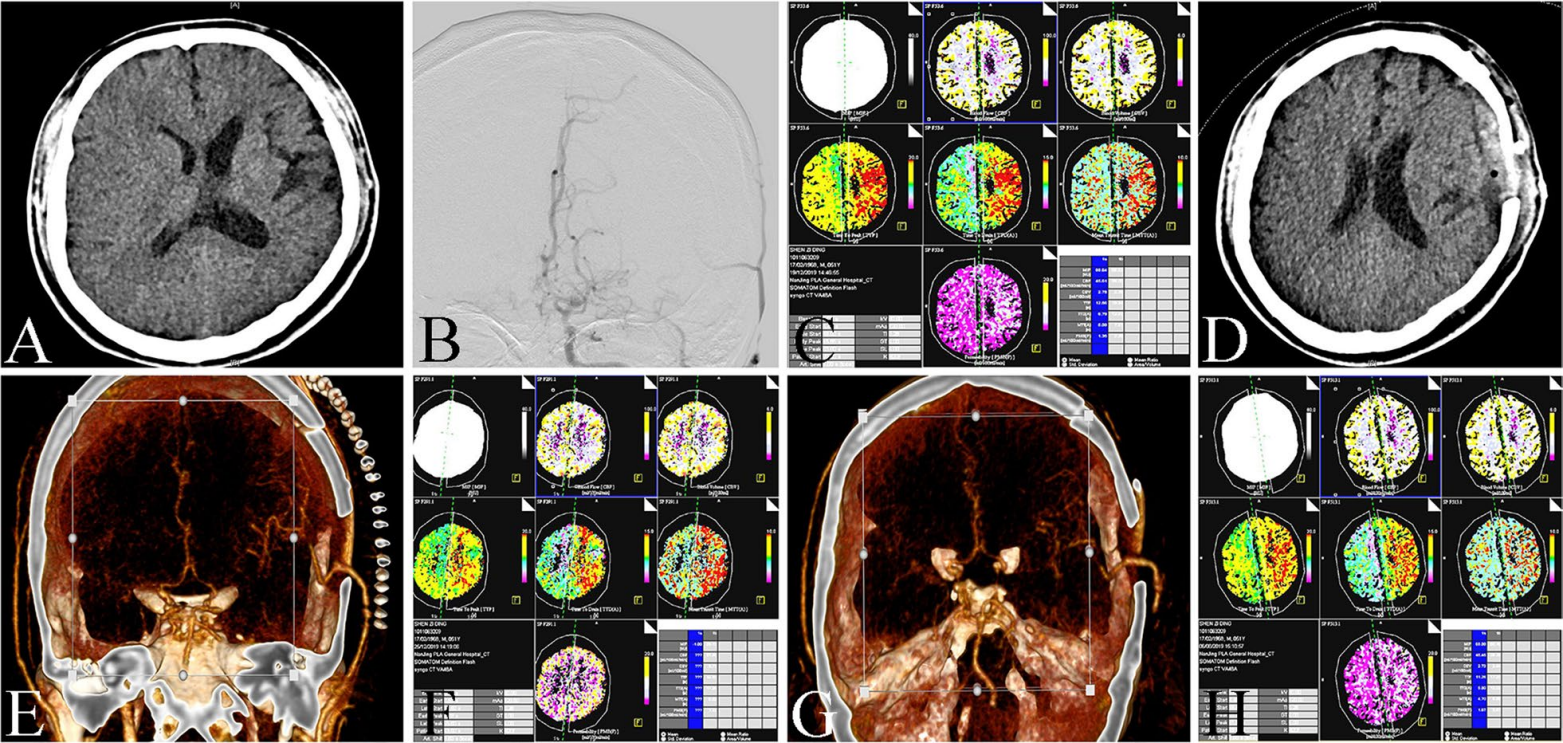
The image data of STA-MCA bypass. **A** Head CT showed infarcts in the left frontal lobe, corona radiata and centrum ovale. **B** DSA showed occlusion in left ICA. **C** CTP showed that the perfusion of left hemisphere was lower than that of right hemisphere. **D** Head CT showed that there was no new infarction or bleeding in the operation area. **E** CTA showed that the bypass vessels were unobstructed. **F** CTP showed that the left hemisphere perfusion was better than that before operation. **G** CTA showed that the bypass vessels were unobstructed after three months of follow-up. **H** CTP showed that left hemisphere perfusion was further improved after three months of follow-up

Head CT was used to evaluate whether there was large area cerebral infarction or internal capsule infarction in the head (Fig. 2A). DSA was used to evaluate the stenosis degree of MCA and STA (Fig. 2B), CTP was used to evaluate the cerebral blood flow perfusion (Fig. 2C). CTP showed that among the 31 patients, the decreased perfusion of 10 patients were single cerebral lobes, 15 patients were multiple cerebral lobes and 6 patients were unilateral basal ganglia. The degree of stenosis was evaluated according to the North American Symptomatic Carotid Endarterectomy Trial (NASCET) standard [6]. The stenosis degree of patients was shown in Table 1.

### Microsurgery

All 31 patients underwent the parietal branch bypass. Head CT showed that there was no new infarction or hemorrhage in the operation area (Fig. 2D). CTA showed that the bypass vessel of 2 patients was obstructed, 29 patients was unobstructed (Fig. 2E). Among the 31 patients, 1 patient had poor healing of incision, which was improved after incision dressing change daily. 3 patients had dysphoria caused by high perfusion, which was improved after olanzapine treatment. 3 patients had neurological dysfunction caused by low perfusion infarction, which was improved after rehydration therapy. 3 patients had improvement of limb muscle strength, 4 patients had improvement of language dysfunction, 6 patients with recurrent TIA had no symptoms after operation. The other patients had no obvious complications, and the clinical symptoms had no obvious change (including 2 patients with bypass vessels obstruction). The preoperative and postoperative mRs score of patients were shown in Table 1. Compared with the preoperative mRs score, the postoperative mRs score showed that the neurological function of patients was significantly improved after operation, and the difference was statistically significant (Table 2).

**Table 2:** Comparison of mRs score between pre-operation and post-operation in 31 patients

Number	Pre-operation	Post-operation	Z value	P value
31	1 (1, 2)	1 (0, 1)	−2.140	0.032

CTP was performed in 31 patients to evaluate cerebral hemodynamics (Fig. 2F). CBF and MTT were measured in decreased perfusion area and corresponding contralateral area. The results showed that the CBF was significantly increased and the MTT was significantly decreased in the lesion side after operation (Table 3), indicating that STA-MCA bypass could improve cerebral blood flow perfusion.

**Table 3:** Comparison of cerebral perfusion between pre-operation and post-operation in 31 patients

Number	Index	Pre-operation	Post-operation	t value	P value
31	CBF	29.65 ± 5.82	32.91 ± 5.69	−6.362	0.000
31	MTT	12.42 ± 2.14	9.39 ± 2.29	5.769	0.000

### Follow-up

Of the 31 patients, 23 patients were performed with clinical and imaging follow-up. 4 patients with complications at discharge were improved, the other patients did not have any ischemic symptoms. The mRs score demonstrated that the neurological function of patients was further improved than that at discharge (Table 4). DSA or CTA showed that the bypass vessel was unobstructed in 23 patients and 13 patients had good collateral compensation (Fig. 2G). CTP suggested that the cerebral blood flow perfusion was further improved than that at discharge (Fig. 2H and Table 5).

**Table 4:** Comparison of mRs score between post-operative and follow-up in 23 patients

Number	Post-operation	Follow-up	Z value	P value
23	1 (0, 1)	1 (0, 1)	−2.000	0.046

**Table 5:** Comparison of cerebral perfusion between post-operative and follow-up in 23 patients

Number	Index	Post-operation	Follow-up	t value	P value
23	CBF	32.43 ± 5.65	33.96 ± 5.27	−4.041	0.001
23	MTT	9.78 ± 2.30	9.35 ± 1.99	2.472	0.022

## Discussion

Yasargil successfully performed STA-MCA bypass for a patient with Marfan’s syndrome complicated with MCA stenosis in 1967, and achieved good results [7]. Since then, many doctors have carried out this operation. However, the role of STA-MCA bypass in patients with MCA stenosis or occlusion was controversial. Previous studies have shown that STA-MCA bypass could not reduce the incidence of ischemic stroke. In addition, compared to drug therapy, STA-MCA bypass had no obvious advantage in improving the symptoms of patients [4]. Therefore, some scholars did not support the application of STA-MCA bypass in patients with MCA stenosis or occlusion. Recently, studies on extracranial-intracranial vascular bypass showed that for symptomatic ischemic stroke patients with vascular stenosis or occlusion, STA-MCA bypass could improve neurological dysfunction, increase cerebral blood flow and reduce the risk of stroke recurrence [5]. The United States carotid artery occlusion surgery randomized trial (COSS) study also showed that STA-MCA bypass could increase the cerebral blood flow and decrease the recurrence of ischemic stroke compared with the drug therapy group [8]. These studies suggested that STA-MCA bypass was safe and effective for patients with cerebrovascular stenosis or occlusion. The results of our study indicated that after STA-MCA bypass, the mRs score significantly decreased, indicating that STA-MCA bypass could improve the clinical symptoms and life ability of patients. Although the clinical symptoms of some patients did not improve, but this part of patients did not have ischemic stroke again. Furthermore, the follow-up results showed that there was no recurrence of stroke, the neurological deficit symptoms were improved and the mRs score was further reduced. The reason might be that STA-MCA bypass reduced the potential risk of stoke recurrence by increasing the blood supply.

At present, the indications of STA-MCA bypass were not clear. The inclusion criteria used in COSS study were [7, 9]: (1) mild to moderate neurological dysfunction caused by TIA or ischemic stroke (modified Barthel Index ≥ 12/20); (2) MCA stenosis or occlusion is consistent with clinical symptoms, and STA is suitable for STA-MCA bypass; (3) positron emission tomography (PET) shows that the ratio of oxygen extraction fraction (OEF) in lesion side to contralateral OEF > 1.13. In our study, we have improved the inclusion criteria and operative strategy. The first was the choice of patients. Patients with MCA stenosis > 70%, poor colonial circulation, objective hemodynamic disorders showed by CTP, recurrent TIA or stroke after maximum dose drug treatment and unsuitability for intravascular interventional therapy were chosen. Besides, for patients with ischemic events, we performed surgery after 1 month. The second was the operative procedure. Before bypass, we used transcranial Doppler (TCD) to select low flow bypass with blood flow of 20–40 ml/min. After bypass, we used TCD to determine whether the bypass vessel was unobstructed and corrected it in time. The third was the operative skill. By improving the vascular anastomosis proficiency (practice repeatedly), we controlled the bypass time at 30 min to avoid the occurrence of ischemic events caused by prolonged vascular blocking. The whole operation time was controlled at 2 h to avoid intraoperative hypotension and cerebral hypoperfusion caused by prolonged anesthesia. The fourth was the drug treatment. We used preoperative oral aspirin, postoperative hypodermic low molecular weight heparin calcium and oral aspirin to avoid thrombosis. Thus, combined with previous literatures and our study, we thought that the surgical indications are based on not only the pathological changes of vascular stenosis or occlusion, but also the clinical features of patients.

The complications of STA-MCA bypass included poor healing of incision, anastomotic bleeding, anastomotic occlusion, high perfusion dysphoria and low perfusion infarction [10]. In our study, 1 patient had poor healing of incision. In this case, the perforating vessels of STA were burned by electrocoagulation, so that the local scalp was in a state of ischemia, which eventually led to scalp necrosis. Therefore, it should pay attention to avoid damaging the scalp blood supply in the process of operation. 1 patient had dysphoria after operation, which might be caused by the sudden increase of cerebral perfusion after bypass, the symptom of dysphoria could be alleviated by oral sedative drugs. 3 patients had neurological dysfunction caused by hypoperfusion infarction, we thought that this may be related to the hypotension during intraoperative anesthesia. Before successful bypass, the blood pressure should not be lower than the preoperative basic blood pressure. After successful bypass, the blood pressure must be maintained to prevent hypotension. 2 patients had anastomotic obstruction after operation. In these 2 patients, anastomotic bleeding was found during the operation and 1–2 stitches were added. Although TCD showed that the anastomosis was unobstructed in the operation, the added stitches may cause anastomotic stricture, then the thrombosis formed and eventually led to the anastomotic obstruction. Therefore, if anastomotic bleeding was found during the operation, it could be firstly covered with gelatin sponge to stop bleeding. Although the anastomosis was obstructed in these 2 patients, CTP showed that the cerebral perfusion was still improved after operation. We thought that the occlusion of anastomosis might be chronic. During this period, the blood supply of lesion side has improved and collateral circulation has developed.

## Conclusion

The STA-MCA bypass for the treatment of MCA stenosis or occlusion patients has already been studied by several authors in the past. However, due to different results of authors, the effects of STA-MCA bypass were controversial. Our study suggested that after improvement of patient selection and operative strategies, STA-MCA bypass can achieve good results for treatment of MCA stenosis or occlusion patients with cerebral hemodynamic disorders, which may not only confirm the significance of STA-MCA bypass but also provide clinical values. There are still some deficiencies in our study, such as the limitations of auxiliary examination equipment, short follow-up time and imperfect evaluation of neurological function. Therefore, the effects of STA-MCA in the treatment of MCA stenosis or occlusion still needs multi-center, large sample and long-term follow-up.

## Data Availability

All data generated or analyzed during this study are included in this article.
